# Stress in Caregivers of Stroke Patients During Rehabilitation: An Observational Study

**DOI:** 10.7759/cureus.37410

**Published:** 2023-04-10

**Authors:** Gracia Sohkhlet, Kavita Thakur, Sudeep I David, Prerna Verma, Vallari Jadav, Johnson S, Deepu Palal, Nirankush Borah, Amitav Banerjee, Sandeep Nallapu

**Affiliations:** 1 Department of Community Medicine, Dr. DY Patil Medical College, Hospital and Research Centre, Dr. DY Patil Vidyapeeth, Pune, IND; 2 Department of Pharmacology, Dr. DY Patil Medical College, Hospital and Research Centre, Dr. DY Patil Vidyapeeth, Pune, IND

**Keywords:** burden, stress, caregiver, rehabilitation, stroke

## Abstract

Introduction: Stroke causes a high burden of death and disability all over the world. The majority of stroke survivors continue to have difficulties, and their families must shoulder a considerable portion of the expenditures of ongoing rehabilitation and long-term care. In India, stroke rehabilitation is still underachieved due to various reasons leading to delay or incomplete recovery of the patients thus adding up more burden on the caregivers. Thus, studying the caregiver burden of stroke rehabilitation will help policymakers tackle this issue faced by our lower economically challenged citizens.

Objectives: The objective is to measure the perceived burden on caregivers during stroke rehabilitation.

Methods and materials: The observational study was conducted by interviewing the stroke survivors’ caregivers and visiting the physiotherapy OPD using the caregiver burden scale/questionnaire.

Results: The study had 76 caregivers, 51.32% were women and 48.68% were men. The average age for caregivers was 42 years and 55 years for patients. The average duration of giving care was six months. The perceived caregiver burden score was low (mean-19.61) suggesting that not all assistance is associated with stress. The correlation of each burden measure with Modified Rankin Scale for disability is significantly correlated (r=0.7, P<0.0001). Further investigation revealed that caregivers had considerably higher levels of stress when the patient needed to exercise, walk or use the restroom. A low yearly income, a higher secondary education, and a small number of family members were shown to be connected with individuals who scored the highest on stress.

Conclusion: Based on this study, we conclude that people with low income residing in nuclear families require more support for caregiving during rehabilitation. We recommend that health and welfare policy measures be developed to lessen caregiver burden in order to improve caregivers' post-stroke experiences.

## Introduction

In India, the stroke prevalence is 1.8% and nearly 40% of them sought rehabilitation services from physical therapy or occupational therapy clinics [1]. According to the Indian Global Burden of Disease Study 1990-2019, stroke was the main cause of death due to neurological illnesses and the leading cause of disability-adjusted life years (DALYs) in India. The factors that substantially influence using rehabilitation services include household economic situation, gender, place of residence, need for additional assistance with daily tasks, and availability of health insurance [1]. Stroke has many social, physical, emotional, occupational, and economic implications on the patients and their caregivers [2].

The existence of other chronic illnesses can make stroke recovery more difficult. Three or more comorbidities are present in up to 75% of stroke survivors, hindering their rehabilitation and necessitating continuous treatment. The most prevalent comorbidity in stroke survivors is hypertension, which is also a risk factor for stroke [3]. Arthritis, asthma, anxiety, mood disorders, hyperlipidemia, and diabetes are other comorbidities that are frequently reported in association with stroke. This often necessitates complex prescription regimens, numerous expert consultations, and more difficult self-care. Due to continuing disability, stroke survivors usually require long-term assistance from their caregivers. The majority of daily care for the stroke survivor is provided by their caregiver, with 61%-91% of them doing so at 12 months after the stroke [3]. Furthermore, caregivers devote a median of 35 hours per week to care for the patient during the first year after a stroke. While helping a stroke survivor on a continuing basis might be gratifying, it can also be harmful to the caregiver's health and well-being like their psychological health (e.g., anxiety, stress), poor social relationships with family and friends), money, and work. As an illustration, long-term (> 6 months) caregiving is linked to higher levels of depression, lower quality of life, physical weariness, anxiety, worse cognitive function, and even mortality in the caregivers [4]. Post discharge from the hospital, care of stroke patients is mainly done by unpaid family members of the p­­­­atient [5]. These members face several challenges while taking care of stroke patients. Therefore, this study was conducted to measure and understand the perceived burden on caregivers during stroke rehabilitation.

## Materials and methods

This observational study was conducted in the physiotherapy department of a tertiary care hospital in Pune, Maharashtra between November 2020 and November 2021. Assuming the prevalence of caregiver burden is 25% [6] and an acceptable difference of 10%, the minimum sample size was calculated as 73 for caregivers using Winpepi software version 2.62.

A total of 76 caregivers were interviewed by convenience sampling, i.e., caregivers who accompanied the stroke patients to Physiotherapy OPD. The family caregivers of stroke patients visiting physiotherapy outpatient or inpatient departments were interviewed using a questionnaire based on the caregiver burden scale and modified Rankin scale for stroke patients to assess their disability [7]. For qualitative assessment, one open-ended question was asked to the caregiver to elaborate on their reasons for stress while taking care of post-stroke patients.

This study was approved by the institutional ethics sub-committee and all those family caregivers of stroke patients who gave consent and were above 18 years of age were included in the study (IRB-I.E.S.C/293/2021).

Definition and details of scales administered

The caregiver is “a person who lives with the patient and is most closely involved in taking care of him/her at home”. A caregiver can also be defined as “an unpaid person who helps with the physical care or coping with the disease” [7].

Caregivers' burden is “the state resulting from necessary caring tasks or restrictions that cause discomfort for the caregiver” [7].

The modified Rankin scale assesses the disability of stroke patients. It evaluates independence as opposed to task performance. There are six ratings on the scale, ranging from 0 (no symptoms) to 5 (severe impairment). For clinical purposes, mild disability runs between 0 and 2, substantial impairment ranges between 3 and 4, and severe disability ranges between 5 and 6 [8].

The caregiver burden scale (CBS) developed by Caroline Macera in 1991 was used to assess the perceived burden among caregivers [9]. CBS is based on 15 internally consistent items (alpha=0.87) useful for assessing perceived stress associated with specific caregiving responsibilities. The quantity of support provided by the carer and the stress that went along with it in 15 areas (1 point each for each area and 3 sub-domains for each, totaling a maximum score of 45) where the patient could need aid were assessed in order to determine the perceived load of the carer (refer to Appendix). The 15 tasks were walking, transportation, housekeeping, farming or yard work, house repairs, bathing, cooking, shopping decision making, financial record keeping, administering medication, dressing, toileting, and leaving the patient alone were all included. One of the key elements affecting a person's or a family's health is their socioeconomic status (SES) for which BG Prasad’s classification (updated May 2021) was used [10].

Data analysis

Data were entered in Microsoft Excel 2019 (Part of Microsoft Office Professional Edition [computer program]. Microsoft; 2019) and analyzed using, Epi Info v7.2 (Dean AG, Arner TG, Sunki GG, Friedman R, Lantinga M, Sangam S, Zubieta JC, Sullivan KM, Brendel KA, Gao Z, Fontaine N, Shu M, Fuller G, Smith DC, Nitschke DA, and Fagan RF. Epi Info™, a database and statistics program for public health professionals. CDC, Atlanta, GA, USA, 2011.) and MedCalc v18.2.1 (MedCalc Statistical Software version 18.2.1 (MedCalc Software, Ostend, Belgium; http://www.medcalc.org; 2018).

Categorical variables were summarized using number (N) & percentages (%) and 95% confidence limits (where applicable), continuous variables expressed as mean and SD & Median, and IQR (where applicable). Normal distribution was verified by the Shapiro-Wilk test. Mann-Whitney U test (where applicable) was used to check for the significance of observations between the two groups. The Kruskal-Wallis test was used to check for the significance of observations between multiple groups. The interviews were transcribed in English. Thematic analysis was done for in-depth Interviews.

## Results

Of the 76 caregivers interviewed, 39 (51.32%) were women and 37 (48.68%) were men. The average ages were 42 years for caregivers and 55 years for patients. About 25% of the caregivers were graduates 19 (25%), 22 (28.95%) were higher secondary, postgraduate 8 (10.53%), primary 4 (5.26%), secondary 5 (6.58%), and senior secondary 18 (23.68%). Among these participants, 85.53% were Hindus, 9.21% were Muslims and 5.29% were others. The average duration of giving care was six months.

About 48.68% of participants were urban and 51.32% were rural. The median annual income of patients was found to be Rs 200,000. There were also some patients who concurrently had comorbidities like diabetes, hypertension, and CVA (Table 1).

**Table 1:** Stroke patient’s comorbidities

Co-morbidities	Frequency (%)
Diabetes mellitus	12 (15.8)
Hypertension + Cerebrovascular accident	1 (1.3)
Hypertension + Diabetes Mellitus + Cerebrovascular accident	1 (1.3)
Hypertension	34 (44.7)
No Comorbidities	28 (36.8)
Total	76 (100)

The patients' requirements and carers' chores were added up, and the results show that these patients have a wide range of demands, with the majority of these needs being met by the carers. The low perceived load score for a small number of actions, however, implies that not all assistance is related to stress (Table 2).

**Table 2:** Caregiver burden score domain wise

Burden domain	Mean (SD)	95% CI	‘P’ value
a. Patient needs assistance*	6.56 (3.86)	5.68 to 7.44	0.0002
b. Do you provide assistance*	8.59 (4.25)	7.62 to 9.56	<0.0001
c. Does it add to your stress level*	4.59(3.60)	3.76 to 5.41	0.0006
Total burden score (a+b+c+d)	19.69(10.71)	17.17 to 22.1	0.0013

**Figure 1:**
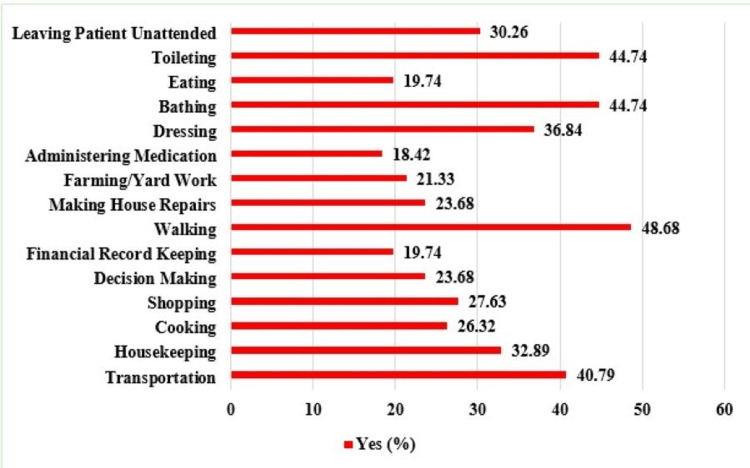
Caregiver stress for different activities of stroke patients

More than half of respondents experienced personal stress related to bringing the patient on walks and to the bathroom, according to the scores for each item (Figure 1).

The correlation of each burden measure was done with Modified Rankin Scale for disability. Total burden score and patient provided with assistance were highly significantly correlated, patient needs help and caregiver stress were moderately significant (Table 3). 

**Table 3:** Modified Rankin Disability score and Caregiver Burden scale correlation Source: Saha I. Essentials of Biostatistics & Research methodology. Published by Academic Publishers. 3rd ed. p. 180.

Burden score	Spearman’s coefficient rank correlation (rho)	95% CI	‘P’ value
Patient needs help	0.69	0.56 to 0.79	<0.0001
Patient provided with assistance	0.74	0.62 to 0.82	<0.0001
Caregiver stress	0.46	0.27 to 0.63	<0.0001
Total burden score	0.70	0.56 to 0.79	<0.0001

It was also observed that there was significance in correlation amongst the caregiver tasks and the patient needs. The caregiver's age or gender had no effect on the perceived burden score. The perceived burden score was significantly higher among caregivers who earned Rs. 50,000 or less (Table 4).

**Table 4:** Caregiver burden score with various variables: *Kruskal-Wallis test        # Mann-Whitney test

Main variable	Variables	n	Median (IQR)	P value
Age	Less than 45 years	14	21 (13-29)	0.36*
	45 – 60 years	35	23 (14.3-29)	
	More than 60 years	27	15 (6.8-28.8)	
Patient Gender	Female	20	21.5 (15-27.5)	0.93#
	Male	56	20 (11-29.5)	
Caregiver Age	Less than 45 years	46	24 (12-30)	0.13*
	45 to 60 years	26	19 (9-26)	
	More than 60 years	4	15 (6.5-23.5)	
Caregiver Gender	Female	39	19 (11.2-26.8)	0.1#
	Male	37	25 (11-31)	
SES (BG Prasad Classification)	1 (Upper)	50	25 (18-30)	0.000012*
	2 (Upper middle)	19	7 (1.3-14.8)	
	3 (Middle)	7	23 (3.5-29.5)	
Education	Graduates and above	26	17.5 (11-29)	0.57#
	Up to Higher Secondary School	50	23 (11-29)	
Type of Family	Joint Family	33	24 (10.8-29)	0.93#
	Nuclear Family	43	19 (12-29)	
Modified Rankin Scale	0	6	25.5 (19-31)	0.11*
	1	15	29 (15-29.8)	
	2	19	22 (15.3-28.8)	
	3	10	15 (4-24)	
	4	16	12 (7.5-22.5)	
	5	10	27 (11-29)	
Completed months of rehabilitation	Less than 1 month	55	21 (12-29)	0.47*
	1 – 6 months	11	15 (5.7-27.2)	
	More than 6 months	10	22.5 (1-29)	
Location	Rural	39	22 (11-29)	0.65*
	Urban	37	19 (11.8-28.2)	
Religion	Hindu	65	21 (11.7-29)	0.33#
	Muslim	7	22 (16-26.5)	
	Others	4	2 (0.5-18.5)	
Marital Status	Unmarried	8	16 (11.5-20.5)	0.24#
	Married	64	23.5 (12-29)	
	Widow	4	9.5 (6-21.5)	

Caregiver’s perspective on stress related to various tasks undertaken by them while taking care of stroke patient is given briefly in Table 5.

**Table 5:** Caregivers' perspective to factors related to stress.

TASKS	VERBATIMS-Caregiver’s perspective on factors for stress
Transportation	“I don’t own a car”
	“Patient is heavy weight so difficult to carry him”
	“I cannot drive her everywhere because I also have to work”
	“He (patient) cannot sit in two-wheeler because of his balance loss. So, I have to borrow my relative’s car.”
	“He (patient) is the only one in the family who knows how to drive. I have to hire auto every time to go for check-up”
Housekeeping	There is so much work at home. I (caregiver) feel very tired at the end of the day.”
	“There is no one at home to help me (patient). So, I had to hire a maid.”
	“My wife is looking after everything including my shop.”
	“I (caregiver) need to do everything myself because we cannot afford to hire help.”
Cooking	“I (caregiver) have to cook two separate meals for my husband and kids. As my husband requires a strict diet”
	“She (patient) used to love to cook, but now I (caregiver) have to cook for her and she is not satisfied.”
	“We have 7 members in our family and I find it very difficult to cook for everyone after mother fell ill.”
	“I (patient) have to depend on my daughter-in-law to cook for me.”
Shopping	“Since we have a baby at home, we have to buy diapers and some days we have to manage without diapers so that we can buy his medicines instead.”
	“I have no one to help me at home to look after her (patient), so I have to request my family members to buy vegetables for us.”
	“Sometimes my neighbours bring groceries.”
	“Elder brother (patient) is already very old. He has to depend on his children for shopping”
	“I do not have time to go to the market. My son-in-law brings groceries.”
Decision making	“My father (patient) is the head of the family in our joint family. Now we have to discuss with the family to make any decision.”
	“I am the only son so I have to make all the decisions now.”
Financial record keeping	“No one else will do it. It is my responsibility now.”
	“I m unsure about maintaining records.”
	“My mother no longer does it, so I (son-in-law) have to do it.”
	“My brother has started managing after father’s illness.”
	“I am stressed because we have low income.”
	“I am paying father’s treatment with a loan.”
	“I do not know how to keep the records. There are so many receipts.”
Walking	“My grandmother cannot walk. Now she has bed sores.”
	“He needs support for walking.”
	“Since she is paralysed, it’s difficult to make her walk.”
	“I am alone with him (patient) so I cannot support him while walking.”
	“I have to wait for my elder brother to help our father.”
	“He is in a wheelchair; it is hard to get him out of the chair to the bed since he cannot walk.”
Making home repairs	“Brother makes the repairs in the house.”
	“There were damages from heavy rain but cannot afford it.”
	“Have to hire someone to help with repairs.”
Farming or yard work	“My mother loved gardening now my sister or maid helps out.”
	“Our garden is left without care.”
	“My uncle (patient) used to do the work but now my cousin is looking after it.”
Administering medication	“There are so many medicines. I get confused.”
	“Sometimes I'm not there so I worry about the medicines.”
	“I worry he might take the wrong medicine.”
	“He is very old. I need to supervise his medicines.”
Dressing	“My husband wants to do everything on his own but he cannot. I have to help him get dressed.”
	“Some has to help her but she does not feel comfortable.”
Bathing	“I am worried he might slip and fall in the bathroom.”
	“She (patient) is very uncooperative and wants a specific water temperature for her bath.”
	“He wants to have bath on his own but I cannot allow that. He needs my help until he has recovered.”
	“My mother-in-law only wants her children to help her.”
Eating	“He drops his plate so often that’s why we have to help him.”
	“She is so picky with her food. We had to change her diet but she does not like it.”
	“My children and I have to take turns to feed him.”
	“Sometimes we have to force feed her.”
Toilet	“It is very difficult at home because he uses a bed pan.”
	“She cannot use an Indian toilet.”
	“There has to be someone to support him because it is slippery inside.”
	“He refuses to take other people’s help. Only wants his wife to help.”
Leaving patient unattended	“Because I have to be with her all the time, I cannot finish my own work.”
	“If I go out somewhere, she feels lonely.”
	“When I have work, I feel guilty to leave him alone.”
	“My mother would cry if I left her alone for long time.”

**Figure 2:**
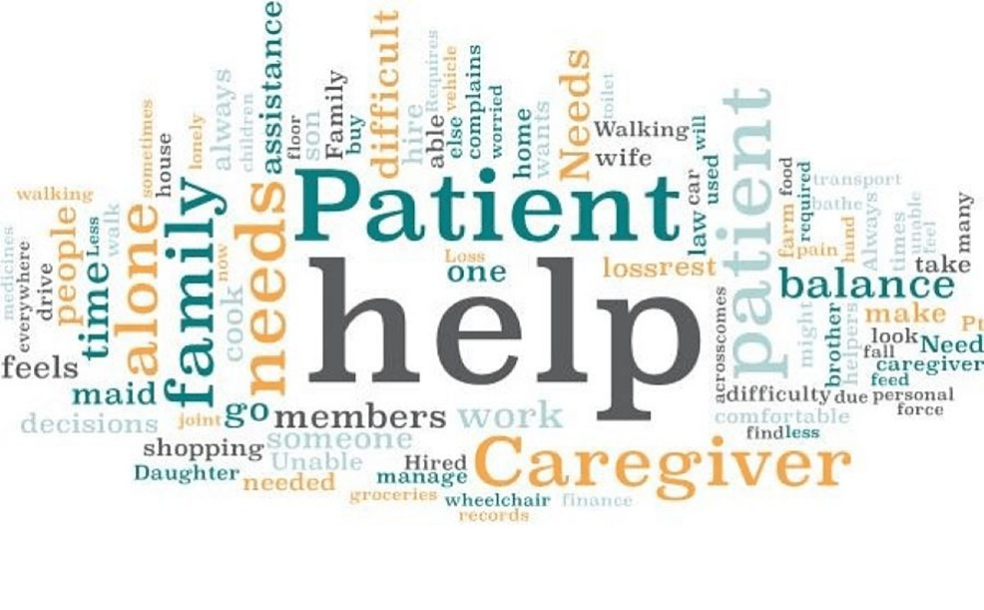
Word cloud for caregivers’ responses to causes of stress.

A word cloud was created to effectively highlight the most frequently used words in the caregiver's verbatims (Figure 2).

## Discussion

In a conventional Indian family, the men provided the family's income while the women looked after the home. The financial burden on the female family members as well as the patient's domestic responsibilities rises when a male stroke patient lives there. Only a few comparable research noted that factors including younger age, female gender, and longer caregiving hours increased the carer’s burden. There was also a significant caregiver burden associated with a decrease in stroke duration, socioeconomic class, and education [10,11].

In this study, we discovered a weak-positive correlation between caregiver burden and patient’s age, level of disability, socioeconomic status, and greater caregiving hours, particularly among female caregivers and male patients. Other studies used a different burden scale like the caregiver reaction assessment (CRA) that provided positive and negative experiences of caregiving [12]. In a study that used the CRA for evaluation of burden, they highlighted that there were caregivers who faced difficulties to pay for the patient’s health needs and services. Others reported that they did not have the strength to care for the patient and were tired most of the time from taking care of their relative. Many shared their experience of eliminating important schedules to be able to meet the patient’s requirements. Caregivers expressed that their burden was highest for bathing, feeding, and helping the patient with the toilet [13]. Our study findings were also similar.

Another study used the caregiver strain index in a cohort study and collected data at six and 12 months post stroke. The perceived burden was consistent over time, however, there was a noticeable change in strain between six and 12 months. They also observed that caregivers living with patients who had a hemorrhagic stroke had a risk of higher burden [14].

Similar to this study, Kavga et al. stated the educational level of a patient, the number of family members living in the same house, the presence of equipment and amenities in the house, and the duration of given care were all connected with a greater sense of burden [15].

In an experimental study by Farahani et al., 116 caregivers of stroke patients were recruited and randomly allocated into two groups where one group received a supportive home care program and the other a routine hospital education program. They concluded that the group with a supportive home care program had comparatively lower burden scores. Hence providing support for home care can help decrease or prevent the burden on caregivers [16]. McCullagh et al. indicated that advanced age and anxiety in stroke patients, excessive reliance, and low family support were predictors of increased caregiver burden [17].

Even though previous studies have highlighted elements that can help caregivers be better prepared for stroke and its effects on care, health planning, recovery, and public health policies seldom take these concerns into account, which lowers participation and increases uncertainty. Therefore, it is important to concentrate on developing recovery-related methods that encourage caregiver engagement [18].

## Conclusions

Stroke is among the leading cause of early death and disability in India, particularly in low- and middle-income groups. It has a tremendous influence on a family's daily life because of the resulting physical and mental difficulties, financial limitations, need for ongoing care, and high prevalence among the elderly population. On the evaluation of the CBS, there were significant findings when correlated with caregiver gender and Prasad's socioeconomic classification, i.e., female caregivers were more of a majority and had more perceived burden, and those who had a lower socioeconomic status and who had up to higher secondary education had a higher score of perceived burden. There was a positive significance of the total scores for patient needs and caregiving assistance, although there was no significance in the stress score; suggesting not all assistance is associated with stress. The perceived burden score did not vary by age or sex of the caregiver. It is imperative that more research be done on techniques and treatments to lessen caregivers’ burden so that structured and focused interventions may be developed by policymakers to lessen their stress.

## Appendices

**Table 6:** Caregiver burden scale

Characteristic	Patient Needs Assistance?	Do You Provide Assistance?	Does it Add to Your Stress Level?	Why?
Transportation	Y N	Y N	Y N	
Housekeeping	Y N	Y N	Y N	
Cooking	Y N	Y N	Y N	
Shopping	Y N	Y N	Y N	
Decision Making	Y N	Y N	Y N	
Financial Record …Keeping	Y N	Y N	Y N	
Walking	Y N	Y N	Y N	
Making House …Repairs	Y N	Y N	Y N	
Farming/Yard …Work	Y N	Y N	Y N	
Administering …Medication	Y N	Y N	Y N	
Dressing	Y N	Y N	Y N	
Bathing	Y N	Y N	Y N	
Eating	Y N	Y N	Y N	
Toileting	Y N	Y N	Y N	
Leaving Patient …Unattended	Y N	Y N	Y N	
